# Multiverse simulation to explore the impact of analytical choices on type I and type II errors in a reaction time study

**DOI:** 10.3758/s13428-025-02807-y

**Published:** 2025-09-18

**Authors:** Miklos Bognar, Marton A. Varga, Don van Ravenzwaaij, Zoltan Kekecs, James A. Grange, Mate Gyurkovics, Balazs Aczel

**Affiliations:** 1 Doctoral School of Psychology, Eötvös Loránd University, Budapest, Hungary; 2 Institute of Psychology, ELTE Eötvös Loránd University, Budapest, Hungary; 3 Department of Psychology, University of Groningen, Groningen, Netherlands; 4 School of Psychology, Keele University, Newcastle, UK; 5 School of Psychology, University of East Anglia, Norwich, UK

**Keywords:** Researchers'degrees of freedom, Simulation, Reaction time, Multiverse, Analytical variability

## Abstract

Researcher degrees of freedom in data analysis present significant challenges in social sciences, where different analytical decisions can lead to varying conclusions. In this work, we propose an example of an exploratory multiverse simulation to empirically compare various decision pathways to identify an effect’s sensitivity to different analytical choices. The approach is demonstrated on the congruency sequence effect (CSE), a well-studied phenomenon in cognitive control research. We reviewed existing literature to identify common non-theory-specific analytical decisions, such as outlier exclusion criteria and hypothesis testing methods, and incorporated these into our simulation framework. Using 20,000 simulated datasets, we compared the true positive rates (TPR) and false positive rates (FPR) across 50 different decision pathways, resulting in a total of 1 million analyses. Our results indicate substantial differences in power and type I error rates across the analytical pathways, with some posing a significant risk of producing high false positives. The findings underscore the importance of running extensive simulations to investigate different data handling and hypothesis testing approaches in certain research fields. This case study serves as an example for conducting similar simulation procedures in research fields characterized by high variability in analytical decisions when investigating an otherwise widely accepted effect.

**Supplementary Information:**

The online version contains supplementary material available at 10.3758/s13428-025-02807-y.

## Introduction

In a typical experimental research project, researchers face many decisions during the analytical process where the choices are not straightforward. Research traditions, different recommendations, and other considerations can implicate different approaches that seem equally viable. These degrees of freedom are hard to navigate, especially for young researchers or scientists new to a research field. In this paper, we propose a form of investigation that can help compare different decision pathways empirically to make grounded choices.

Researcher degrees of freedom (Simmons et al., 2011) are well-known phenomena in social sciences. The result of high researcher degrees of freedom is that different choices during data collection, data preparation, and data analysis can lead to entirely different conclusions. The meta-scientific literature around the replication crisis often criticizes the practice of choosing only one path in the analytical decision tree and not disclosing the rationale of these choices (Gelman & Loken, 2013). One can argue that doing so might raise the suspicion that a certain choice in data preparation and analysis results from a questionable research practice aiming to pick a path that results in a favorable conclusion for the researcher (Anvari & Lakens, 2018). Of course, specific decision paths in data analysis are not necessarily the product of opportunistically cherry-picking favorable results. In most research areas, there are long-standing traditions in analytical approaches that are considered standard or best practice, given how commonly they emerge in the literature. However, when several analytical decision pathways are available for the same hypothesis, in the absence of empirical evidence favoring one option over the others, the norm is to choose one pathway arbitrarily.

In response to the problem of researcher degrees of freedom, several new approaches have been suggested to compensate for the distorting effect of analytical variability on global conclusions around an effect. In a multi-analyst approach (Aczel et al., 2021), multiple independent researchers are asked to analyze the same dataset using the pipeline of their choice to see how analyst decisions affect the results of a study. Another approach to addressing analytical variability is the multiverse analysis method, where all potential decision paths in data preparation and analysis are taken to provide a wide array of results regarding the same research question (Steegen et al., 2016).

Analyzing data along all possible pathways might provide a broader picture of the sensitivity of the analysis to alternative choices. However, it is important to define analysis steps that are strictly linked to theory and are indispensable to addressing the effect of interest correctly. For example, in a study where a hypothesis about the effect of cognitive conflict on correct response time is tested, excluding erroneous trials from the analyzed dataset is obligatory. Aside from analytical choices that are linked to theory, some choices are not directly linked to the investigated theory but are taken based on historical traditions in the field or researcher habit. For example, most theories in psychology do not specify participant exclusion criteria (e.g., attention check methods), a plausible range along the distribution of the dependent variable that the investigated effect is expected to be, or the hypothesis testing method required to find the effect. This flexibility in theories gives researchers more degrees of freedom, which is often tackled by following previous practices in such decisions. One way to plan a decision tree for a multiverse analysis is along these “non-theory-based” decision nodes, where the choices are arbitrary or their impact on the effect is uncertain (Del Giudice & Gangestad, 2021).

In all confirmatory studies, it is helpful for researchers to prepare an analysis plan before the experimental phase. Both in preregistrations (Nosek et al., 2019) and in registered reports (Chambers, 2013; Nosek & Lakens, 2014), preparation and reporting of data analysis decisions is a key step before conducting the study. These preemptive decisions are considered to improve the credibility of findings through a broad consensus in the scientific community. While it is helpful to prepare and report analytical decisions, registered analysis plans often do not provide rationales for the analytical choices. The same problem applies to multiverse studies, where differences between the results of various analysis techniques can be assessed only in one particular study. Still, multiverse results cannot inform the research field about which of these techniques would be appropriate for future studies.

In a large-scale multi-analyst reaction time (RT) study, Dutilh and colleagues (2019) tested how different researcher teams estimated the value of psychological constructs (e.g., response caution or difficulty). The study manipulated different conditions in the experiment, and analysis teams were provided with the data, but they were blind to which conditions were manipulated. Their goal was to accurately estimate how conditions were manipulated. Most importantly, all 17 teams chose different procedures—all considered to be standard approaches—to draw their inferences. The inferences were also considerably different from each other. While this study aimed to highlight the importance of cognitive models in RT research, the results show that many different approaches can be acceptable simultaneously, but they may yield variable results. However, such variability raises the question of whether one analytical approach is better or worse than the others: how can we identify the benefits of using one analysis technique over another in a particular area?

Without preliminary exploration, it is unclear how analytical decisions affect the discoverability of a newly reported effect. Most studies, however, build their theories on base effects that are well established and widely considered as existing phenomena within a given research community. Many psychological effects, such as the Stroop effect (Stroop, 1935) and other congruency effects (Eriksen & Eriksen, 1974; Simon, 1990), the list-wide proportion congruency effect (Logan & Zbrodoff, 1979), the post-error slowing effect (e.g., Allan Cheyne et al., 2009), or the congruency sequence effect (CSE; Gratton et al., 1992) demonstrated in this case study, are often used as a basis for more complex hypotheses that predict the modulation, triggering, or cancellation of these base effects due to other factors.

In these complex hypotheses, the base effect is used as the “ground truth”: the assumption is that without further manipulation, the base effect always exists when elicited correctly; the question is only whether it can be manipulated by additional parameters. If there is no clear consensus among researchers in a particular area on what is considered a correct analytical pathway to look for an effect, then multiple pathways are considered equally viable options. We argue that a research community around a “ground truth” effect should establish consensus about which analytical decisions are best for capturing such base effects if they exist—and thus provide the highest likelihood of detecting more complex interactions involving those effects.

There are many ways to compare the performance of different decision pathways: In the present study, we measured the true positive rate (TPR) and the false positive rate (FPR) to quantify the probability of finding an effect when it exists and when it does not, respectively, in simulated scenarios where the ground truth is known. Similarly to the signal detection theory (Pastore & Scheirer, 1974) often used in medical research (Swets, 1979), we considered an analysis better with higher TPRs and lower FPRs.

In this study, we provide an exploratory example within which different analytical decision pathways can be compared. In doing so, we focus our attention in particular on data analysis. Methodological discussions are usually part of theory development, and experimental methods are explicitly considered when formulating a theory about how an effect works. Apart from a few exceptions, however, most analytical steps are not specified in theories. For example, the Stroop effect is a well-known reaction time phenomenon, and many theories have discussed it in great detail (Botvinick et al., 2001; Cohen et al., 1990; Posner & Snyder, 1975; Stroop, 1935), outlining several methodological steps (e.g., target and distractor sets, stimulus duration, response sets) and a few analytical steps (e.g., excluding error trials in a Stroop task when analyzing reaction times).

These methodological and analytical steps are explicitly discussed in theories because they are necessary in order to exclude confounders or alternative explanations of the effect. However, there are also several analytical steps—such as outlier filtering or hypothesis testing models—that are not specified in such theories. From here on, we label these two categories “theory-specific” and “non-theory-specific” analytical choices, respectively. We hypothesize that the latter can still influence the outcome of studies without introducing confounds or alternative theoretical explanations, simply by emphasizing different features of the underlying distribution of the dependent variable. Our proposed framework seeks to detect how these non-theory-specific analytical steps might alter the discoverability of a “ground truth” effect, an effect that is widely accepted among researchers but lacks consensus of its analytical measurement. It is also important to assess whether different—otherwise common-practice—data handling or hypothesis testing approaches can alter distributions in ways that may lead to unnoticed false positives. Different analytical pathways may show optimal sensitivity at different sample sizes; thus, the available participant pool is also a crucial variable in deciding which pathway is best to use.

To demonstrate this exploratory method, we utilize it on a well-known phenomenon often attributed to cognitive control, the congruency sequence effect (CSE; Gratton et al., 1992). An exploratory multiverse simulation might help find the most efficient, or least biased, way to analyze experimental datasets on well-established effects more generally and provide a rationale for analytical decisions that are usually not part of the effect’s literature. This multiverse simulation consists of outlining a decision tree of interest and analyzing simulated data along all pathways. By creating a decision tree and testing a large number of simulated datasets either containing or not containing the effect of interest on all paths, we will be able to compare TPRs and FPRs between different analytical decisions. If diagnostics vary across different pathways, researchers should be informed about how analytical decisions affect TPR and FPR, and which analytical pathways are best to use or best to avoid. In this work, we offer a case study that can be utilized in different research areas to establish empirical insights into how sensitive specific effects are to analytical decisions. The scale of implementation can vary, and with a large enough scale—as demonstrated in this study—general conclusions can be made based on it to universally inform future researchers of a specific area of research. However, on a smaller scale, sensitivity differences between small amounts of analytical pathways can also be investigated by individual researchers before conducting confirmatory studies.

### A case study: Congruency sequence effect (CSE)

Cognitive control refers to the set of mechanisms that help us choose and execute behaviors in a goal-directed way, often in contrast with otherwise automatic behaviors (Cohen, 2017). Various subprocesses of cognitive control are investigated through different behavioral tasks (von Bastian et al., 2020), such as interference tasks, where participants are asked to respond to sets of stimuli that induce different levels of cognitive conflict. For example, in a flanker task, an array of stimuli is shown to participants (e.g., letters or arrows), and one should respond to the middle stimulus while ignoring “flanking” stimuli on the side. On incongruent trials, the central stimulus and flanker stimuli are different, leading to a higher processing load due to increased conflict compared to congruent trials, where the central and lateral stimuli prime the same response. This creates a difference in task performance, the so-called congruency effect (calculated as congruent performance subtracted from incongruent performance), or flanker effect (Eriksen & Eriksen, 1974). A common way to make inferences about the workings of cognitive control is to measure conditional adjustments in the congruency effect (e.g., Braem et al., 2019).

In the last three decades, one of the most commonly investigated conditional adjustments was the congruency sequence effect (CSE), where the congruency effect is smaller when the preceding trial is incongruent than when the preceding trial is congruent. While it is usually not specified, the reduced congruency effect is often the joint result of increased performance on incongruent trials and decreased performance on congruent trials when the previous trial is incongruent (Egner, 2007). This effect can be measured by the RT difference between the four congruency conditions, a factorial combination of the following two factors: previous trial congruent or incongruent (*c* or *i*); current trial congruent or incongruent (*C* or *I*). The following formula can describe the raw effect size of this congruency effect adjustment:

$$cI-cC-\left(iI-iC\right)$$

Over the past decades, several theories have discussed the underlying mechanisms behind the effect (Botvinick et al., 2001; Dreisbach & Fischer, 2012; Mayr et al., 2003; Verguts & Notebaert, 2009), and a plethora of experimental investigations have been conducted to prove or disprove specific theories by measuring CSE. There is broad consensus on the existence of CSE. However, the effect size is relatively small; therefore, a fair amount of statistical power is needed to detect CSE by itself, not to mention more complex designs where higher-order interactions involving the effect are investigated. Over the years, researchers have followed similar designs to detect the effect, and recommendations in design choices (which we would consider theory-specific choices) have also been made to facilitate the minimization of confounding effects and maximization of the chance of finding the effect (Braem et al., 2019; Weissman et al., 2014).

To explore the variability in analytical decisions in CSE-related studies, we conducted a small-scale systematic review of papers published from 2014 to the present day (January 2025). We searched the PubMed database to find papers that mentioned “congruency sequence effect” and were publicly accessible (more details on the literature review in the Methods section).

Although the experimental designs were similar, there is considerable heterogeneity in decisions concerning data analysis in CSE studies. We reviewed a wide array of studies (see Supplementary Table 1) that measured CSE and identified critical decisions throughout the analytical pathways that are not strictly defined by theories explaining CSE and are somewhat arbitrary. The two most important decision nodes were the following:

- Excluding outliers

In reaction time (RT) studies*,* it is common practice to identify and exclude outlier trials where responses are too slow or too fast. While outlier exclusions are ubiquitous in CSE studies, there is no clear consensus on how strict the outlier removal criteria should be and virtually no discussion on what theoretical implications these choices rely on. Several suggestions have been made on RT outliers (Berger & Kiefer, 2021; Boag et al., 2024; Miller, 1991; Ulrich & Miller, 1994); however, there is no definite consensus about which exclusion criteria should be used in CSE studies. In the last decade, a cutoff criterion of ±3 standard deviations (±3SD) from the mean (SD) cutoff criterion has been the most popular approach (34 out of 65 reviewed studies); however, there are several instances where 2.5SD or 2SD cutoffs, or more rigid predefined truncation points, such as thresholds of 1,000- or 1,500 ms, are implemented. The use of median absolute deviation (MAD) is also a recommended practice (Leys et al., 2013), although less popular than the above methods. Berger and Kiefer (2021) have shown that using 2SD or 3SD cutoff points introduces the smallest amount of bias in *t*-test results in simulated RTs compared to other outlier exclusion methods or no exclusions.

- Hypothesis testing models

The hypothesis tests used to investigate CSE are varied, but the most popular approach (used in approximately 86% of all studies) is the repeated-measures analysis of variance (rmANOVA). The repeated-measures approach allows for variability between subjects while comparing conditional means. Another popular approach is linear mixed-effects models (LMM), where trial-level (i.e., non-aggregated) RTs are predicted by fixed effects, such as the CSE interaction, and random effects, such as intercept for participants and different random slope structures. There is significant variability in regression approaches, as some configure only random intercepts, and some prescribe maximal random-effect structures. Some of the LMM approaches used raw RTs as the dependent variable, while others transformed RTs in order to normalize their distribution either with log transformation or with inverse transformation. A rare analysis approach is fitting generalized linear mixed-effects models, where RTs are fitted with an inverse Gaussian distribution with a log link function, thus compensating for the skewed distribution of RTs.

Linear mixed-effects regression approaches allow for analyzing all trials without aggregating them, while controlling for between-subject variability with high flexibility, allowing for random effects for both intercepts and slopes on different levels (van Doorn et al., 2023). Linear mixed model proponents argue that among other advantages, such as robustness towards missing values, the underlying random effects (e.g., between-subject variability) in experimental designs are so vast that linear mixed models with maximal random-effect structures are the most appropriate way to analyze psychological data (Barr et al., 2013). Although more and more arguments against ANOVA and in favor of mixed models are published, mixed models have their disadvantages. Mixed models usually require a large amount of data to fit well, and it is not yet established what the extent of robustness gained from using mixed models in CSE studies is and whether ANOVAs could perform similarly in low-data scenarios.

We quantify the effect of these decisions on power (or TPR) and false positive findings (or FPR). In this study showcasing an example of the application of our framework, our main interest regarding CSE was how different outlier exclusion methods and statistical analysis approaches affect the discoverability of CSE. To answer this question, we tested a large number of decision combinations on simulated CSE data in a multiverse manner.

## Methods

**Fig. 1:**
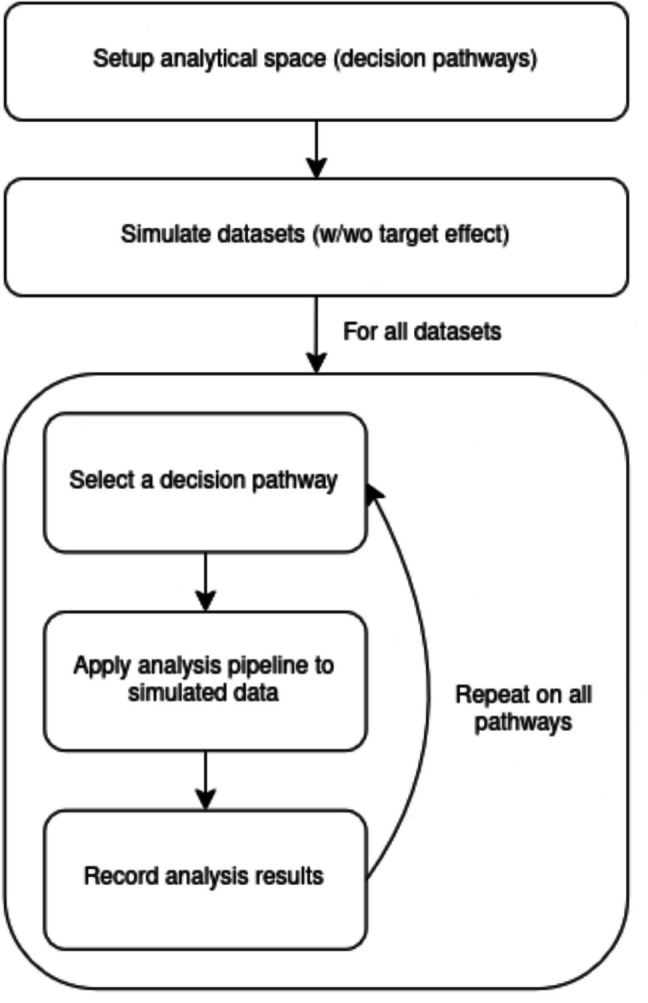
Steps of simulating a multiverse exploration of an analytical space

The exploratory multiverse simulation for an analytical space in RTs demonstrated in this study consists of the steps described in Fig. 1.

### Defining decision pathways of interest

To identify the specific pathways in the multiverse analysis structure that we compare, we conducted a small-scale literature review using the PubMed database of published papers. We searched for papers with the following search tags: *“congruency sequence effect” AND (“reaction time” OR “response time”).*

We included all papers that the above search function found in the review, if it was publicly accessible, and CSE was measured in response time. The list of papers reviewed by this method is far from exhaustive, although it reflects general directions in analytical decisions in CSE research. The reviewed articles are shown in the supplementary materials (Supplementary Table 1).

Based on our review, we identified two major decision nodes (outlier exclusion criteria and hypothesis testing models) and the most common decisions taken within them:

Outlier exclusion strategies:

1. Exclusion based on standard deviation (SD) from conditional mean (z-score):
  ○ mean ± 2 SD
  ○ mean ± 2.5 SD
  ○ mean ± 3 SD

2. Exclusion based on median absolute deviation (MAD) from conditional median:
  ○ median ± 2 MAD
  ○ median ± 2.5 MAD
  ○ median ± 3 MAD

3. Exclusion based on fixed RT bounds (outside range):
  ○ 200–1,000 ms
  ○ 200–1,250 ms
  ○ 200–1,500 ms

4. No RT-based exclusion

We evaluated five hypothesis testing methods:

1. Repeated-measures ANOVA (rmANOVA)
2. Linear mixed-effects model (LMM) with participant as a random intercept
3. LMM with participant as a random intercept and current trial congruency as a random slope
4. LMM on log-transformed RTs with participant as a random intercept
5. LMM on log-transformed RTs with participant as a random intercept and current trial congruency as a random slope

### Simulating RT data

#### Obtained empirical data

As the basis of our simulation, we used data from Bognar et al. (2023), in which flanker and prime-probe tasks were administered online. The design of the two experiments was based on Weissman et al. (2014). The estimated CSE was stronger in the prime-probe dataset compared to the flanker dataset (*prime-probe estimate =* −20.86 ms, flanker estimate = −14.62 ms). Practice trials and every first trial of experiment blocks were excluded from the raw datasets. Due to a software error, the flanker experiment sometimes allowed very long response times. A total of 492 out of 135,360 (0.4%) trials that were more than 4 s long were removed from the flanker dataset. No other data filtering or data exclusion was undertaken on the two obtained datasets.

#### Fitting linear models on obtained empirical data

To estimate fixed and random parameters of CSE effects, we fitted linear mixed-effects models to the two obtained empirical datasets. The most theoretically aligned models with no singular fit on both datasets were models that consisted of a *current trial congruency* fixed effect, *previous trial congruency* fixed effect, a previous trial congruency * current trial congruency fixed interaction effect, *participant* as random intercept, and *current trial congruency* as random slope.

#### Sample sizes

Data simulation techniques are especially useful for analyzing power for more complex models, such as linear mixed-effects models (DeBruine & Barr, 2021). Although we were primarily interested in the power changes depending on analytical choices, to see a more comprehensive picture, we defined five different sample sizes based on the reviewed literature and previous findings. Most CSE lab experiments tend to consist of 25–50 participants, and they usually yield comparatively large effect sizes. While online CSE experiments proved to be efficient in finding CSE, they tended to yield comparatively small effect sizes (Bognar et al., 2023; Gyurkovics et al., 2020; Weissman et al., 2014); thus, higher sample sizes (100-–400 participants) are required to compensate for collecting data online. Therefore, we generated data with 25, 50, 100, 200, and 400 participants.

#### Raw effect sizes

The flanker and prime-probe datasets yielded different estimates for the congruency sequence effect (CSE), with the former showing a smaller effect than the latter. Based on these empirical differences, we defined four types of *true effect sizes* for our simulations and generated data based on these true effect sizes:

- Small effect size: Simulated using the fitted parameters from the empirical flanker RT model.
- Large effect size: Simulated using the fitted parameters from the empirical prime-probe RT model.
- Zero effect sizes: Simulated using the flanker and prime-probe RT model parameters, but with the *interaction term* (previous trial congruency × current trial congruency) set to zero. Two variants of zero-effect datasets were generated for robustness: one based on the flanker RT model, and the other on the prime-probe RT model.

The small and large true effect size models were used to generate true RT congruency sequence effects and accuracy data in participants for the two different tasks, while the zero effect size models were used to generate participant data where CSEs were not present, only a current *trial congruency* main effect.

#### Simulating participant-level effects

To generate the datasets representing the different true effect sizes, we fitted six models to the empirical data:

For both the small and large true effect sizes (based on flanker and prime-probe data, respectively), we used two models, a linear mixed-effects model for response times (RT), and a generalized linear mixed-effects model for accuracy. Both models included a fixed effect for current trial congruency, previous trial congruency, and the interaction of trial congruency and previous trial congruency. Random effects consisted of participant as a random intercept and trial congruency as a random slope.

For the zero true effect sizes, we used the flanker and prime–probe models but applied a permutation-based nullification procedure to disrupt the interaction effect while preserving other effect structures. In this procedure, we randomly swapped data in a way that all the RT main effects were preserved, except the CSE interaction, that was nullified. For the details of the nullification procedure, see the supplementary materials. Briefly, the effect of interest in the model was shuffled to disrupt the connection with RT, then a new model on the disrupted data was fitted. The RT model included a fixed effect for current trial congruency, a fixed effect for previous trial congruency, and a fixed interaction term between current trial congruency and previous trial congruency (nullified via permutation). Random factors included participant as a random intercept, and current trial congruency as a random slope. For accuracy data in the zero true effect size dataset simulations, we used the parameters of the true effect accuracy models.

Simulation parameters were derived from the fixed-effects and random-effects covariance matrices, and residual variance of the originally fitted models to the obtained prime-probe and flanker data. Participant-level random effects for simulations were sampled from multivariate normal distributions based on these covariance matrices. Trial-level RTs were computed as a combination of fixed effects, random effects, and Gaussian noise based on the empirical models. Accuracy was simulated with probabilities determined by fixed and random effects of the accuracy model, then sampling from a binomial distribution based on predicted probabilities. For further details on the fixed and random effects, see the supplementary materials. These RTs and accuracies were then aggregated into means and variances for participants in order to pipe them into a drift–diffusion model.

#### Trial generation with diffusion models

As the participant means simulated in the above step followed a Gaussian distribution, we added an additional layer to the simulation pipeline by using a drift–diffusion model to simulate individual trial RTs for every participants on all four conditions. The drift–diffusion model is a cognitive model originally designed for two-choice experimental designs to model the speed–accuracy trade-off in RTs (Ratcliff, 1978; Voss et al., 2013). The model generates realistically skewed RT distributions and accuracies based on a few parameters, such as drift rate (reflecting processing speed), boundary separation (reflecting response caution), and non-decision time (reflecting that part of the response time not related to the decision, such as stimulus encoding and the motor response).

Based on the participant condition data in the previously generated datasets, with the help of the EZ package (van Ravenzwaaij et al., 2017; Wagenmakers et al., 2007), we transformed the RT means, RT variances, and accuracy percentages for every participant in all four conditions to drift rate, boundary separation, and non-decision time parameters. These diffusion model parameters were then supplied to the “rdiffusion” function of the “rtdists” R package (Singmann et al., 2018) to generate trial-level data. A total of 100 trials were generated per condition per participant (400 per participant).

#### Reaction time contaminants

To simulate situations where participants do not pay attention (e.g., due to mind-wandering or external distractions), we introduced contaminants into the trials. Based on the procedure used in Ratcliff and Tuerlinckx (2002), we exchanged 5% of trials per condition per participant to reaction time data coming not from the drift diffusion model but a random uniform distribution with a minimum of 0 and a maximum of 3.09 s (the range in our empirical flanker dataset). This resulted in a proportion of trials that did not depend on the simulated process informed by participant-level parameters but a random number generator, introducing the possibility to generate both fast and slow outliers.

#### Data simulation summary

To generate highly realistic datasets, we first obtained data from empirical CSE results. By fitting mixed-effects models to the obtained data, we extracted fixed- and random-effect parameters that were then used to simulate participant-level fixed and random effects, which were then, in turn, used to generate trial-level data for all four conditions (*cC*, *cI*, *iC*, *iI*) within each participant. Aggregating the generated trial-level data per participant resulted in four conditional means and variances for every individual. With the use of the drift–diffusion model, a second round of trial-level data simulation was run based on these participant-level means and variances. Finally, 5% of trial reaction times were later exchanged with random numbers between 0 and 3.09 s to simulate contaminant reaction times. These trial-level data were then analyzed.

A total of 1,000 datasets were generated for four dataset types (small effect size based on flanker empirical model, large effect size based on prime-probe empirical model, and two zero effect sizes coming from the nullified flanker and prime-probe empirical models), in five different sample sizes (25, 50, 100, 200, and 400 participants), resulting in a total of 20,000 different datasets. In all datasets, 400 trials were generated for every participant (100 per condition), resulting in a minimum of 10,000 and a maximum of 160,000 trials per dataset.

### Analyzing simulated data

The multiverse decision tree was constructed based on the above-described literature review. We selected two analytical decision nodes with multiple choices: outlier detection and filtering decisions, and hypothesis testing model decisions. The 10 outlier detection choices and the five hypothesis testing models resulted in 50 different decision pathways. All 50 decision pathways were applied to all 20,000 datasets, resulting in 1,000,000 different data analyses conducted. Below, we demonstrate through one dataset how the different analytic decisions were implemented.

#### Data preprocessing

We conducted data preprocessing operations on the generated data according to the decision pathways of interest. First, on all paths, we excluded trials that were error trials, as this is standard practice in published CSE RT analyses (Braem et al., 2019). Then, one of the 10 outlier detection and filtering methods was implemented.

#### Hypothesis testing models

In all reviewed studies, a statistical hypothesis testing method to detect CSE was implemented. Most studies tested not only the existence of CSE but the effect of other between- or within-subject independent variables. CSE in all approaches was tested by hypothesizing a negative interaction (congruency effects smaller when the previous trial is incongruent, compared to when the previous trial is congruent) between the previous and the current trial’s congruency. In most studies, the alternative hypothesis of CSE was accepted when the interaction’s direction was consistent with the hypothesis, and the interaction was statistically significant (alpha =.05). Based on the literature review, we defined five hypothesis testing models to test the above hypothesis.

- *Repeated-measures ANOVA:* The most popular way to detect CSE is a simple 2× 2 repeated-measures ANOVA where RT is predicted by the congruency of the current and the previous trial. To use this hypothesis testing model, we aggregated data by averaging RTs per condition for every participant. We accepted the alternative hypothesis of CSE if the previous and current trial interaction was statistically significant and the interaction’s direction was congruent with the hypothesis.
- *Simple linear mixed-effects model:* This mixed-effects model consisted of a previous trial congruency and current trial congruency interaction fixed factor, and participants only as the random intercept factor. Formula:
  $$RT\sim current\ trial\ congruency\times previous\ trial\ congruency+\left(1\left|paticipant\right.\right)$$
- *Complex linear mixed-effects model:* A more complex model was also tested in which the fixed factor was the CSE interaction, and the random factors consisted of random intercepts for participants and random slopes for current trial congruency. Note that more complex random-effect structures could have been tested; however, during the simulation phase, this random-effect structure was the most complex that could converge on the obtained data, and therefore only these random-effect parameters were specified for the data simulations. Formula
  $$RT\sim current\ trial\ congruency\times previous\ trial\ congruency+\left(1+current\ trial\ congruency\left|paticipant\right.\right)$$:
- *Simple linear mixed-effects regression model on log-transformed RTs:* Due to the skewed nature of reaction time data, many studies use hypothesis testing models that aim to control for this non-normality. We tested a mixed-effects linear regression model fitted to log-transformed RT data. The same fixed and random factor structure was used as in the simple mixed-effects linear regression model: only random intercepts for participants. Formula:
  $$\mathit{log}\left(RT\right)\sim\ current\ trial\ congruency\times previous\ trial\ congruency+\left(1\left|paticipant\right.\right)$$
- *Complex linear mixed-effects regression model on log-transformed RTs*: The same linear regression model on log-transformed RTs, with a complex random-effect structure, was also tested, where random intercepts per participant and random slope for trial congruency were used. Formula:
  $$\mathit{log}\left(RT\right)\sim\ current\ trial\ congruency\times previous\ trial\ congruency+\left(1+current\ trial\ congruency\left|paticipant\right.\right)$$

To conduct hypothesis testing with mixed-effects models for all linear models, we created a null model, where the same fixed and random-effect structures were kept except for the CSE interaction, which was not part of the model. This way, by comparing null models and alternative models with a likelihood ratio test (LRT), we could statistically test whether the CSE was present. We accepted the alternative hypothesis test of CSE if the following criteria were met:

- Akaike information criterion (AIC) difference of null and alternative model was larger than or equal to 2, or Bayesian information criterion (BIC) difference was larger than or equal to 6.
- The LRT resulted in a statistically significant difference between the models.
- The interaction term of the alternative model was statistically significant.
- The predicted direction of the interaction was consistent with the hypothesis.

#### Analysis output

A total of 20,000 datasets were generated for the different effect (large effect, small effect, “large” no-effect, “small” no-effect) and sample size (25, 50, 100, 200, 400) combinations, 1,000 datasets for each. All datasets were analyzed on all 50 pathways, resulting in 1,000,000 different analyses conducted. For each run in a decision pathway, we determined whether the alternative hypothesis was supported (coded as 1) or not (coded as 0) based on the criteria outlined in the previous subsection. Based on these values, we could summarize a decision pathway’s TPR on the two true-effect dataset types, and FPR on the two no-effect dataset types. We expected TPR to increase with statistical power on all true effect pathways and FPR to remain around.025 (slightly lower for the linear models due to the additional inference criteria) based on the predefined alpha level and additional directional criteria in analyses.

## Results

### Data preprocessing

We excluded all model fits with any convergence error from TPR calculations for decision pathways, regardless of the nature of the convergence error. In an experimental study, one might consider different approaches to troubleshoot convergence errors. For clarity of this simulation study, we omitted every model results that outputted any errors for convergence. The percentage of simulations for which models converged per dataset per model are shown in Table 1.

**Table 1:** Percentage of converged linear models on different datasets

Complex LMM	Complex LMM log(RT)	Simple LMM	Simple LMM log(RT)	Dataset
99.84%	99.98%	99.98%	100%	Prime-probe
99.83%	99.95%	99.95%	100%	Flanker
99.83%	99.99%	99.97%	100%	Flanker no effect
99.87%	99.99%	99.94%	100%	Prime probe no effect

### Prime-probe (large-effect) datasets

#### Hypothesis testing models

**Fig. 2:**
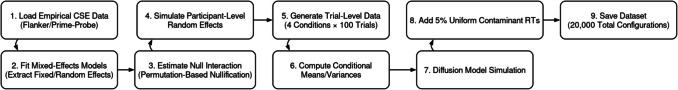
Steps for simulating CSE data in different effect and sample size configurations

**Fig. 3:**
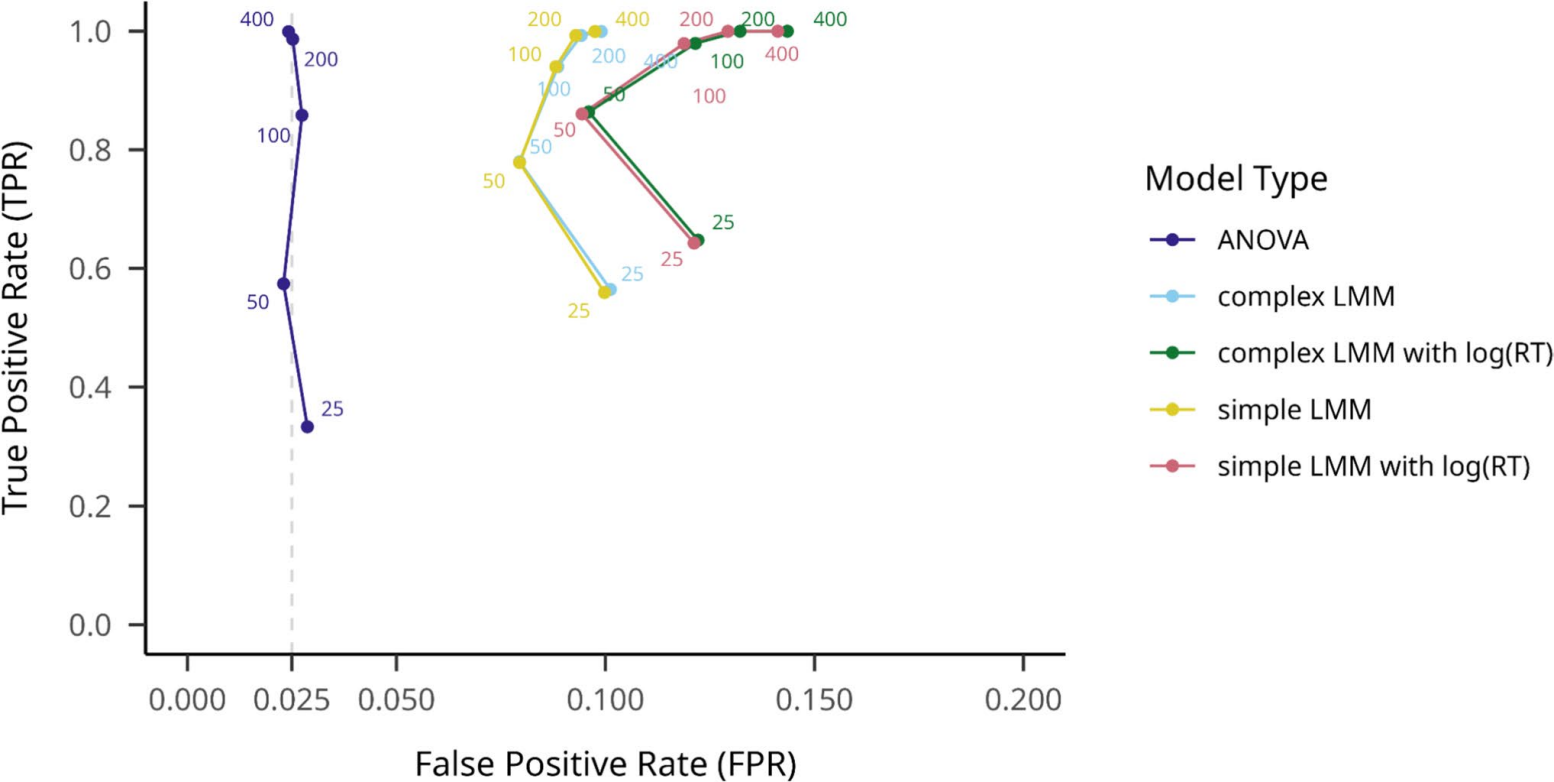
Model TPRs and FPRs on large effect size datasets on different participant numbers. Note. True positive rate is indicated on the *y*-axis, while false positive rate is indicated on the *x*-axis. Hypothesis testing models are shown with different colors, and numbers on the plot indicate different sample sizes. An assumed maximum FPR of.025 is indicated with a dashed vertical line

We aggregated all outlier filtering methods in order to measure average TPR across different hypothesis testing models. As shown in Figs. 2 and 3, overall, the complex mixed-effects linear model with log-transformed RTs obtained the highest mean TPR across participant numbers,.898 (95% CI [.896,.901]), with a rather high mean FPR of.123 (95% CI [.120,.126]), while the repeated-measures ANOVA achieved the lowest TPR, at.750 (95% CI [.747,.754]), combined with an FPR of.026 (95% CI [.024,.027]). The simple mixed-effects linear model with log-transformed RTs reached a mean TPR of.896 (95% CI [.893,.899]) and mean FPR of.121 (95% CI [.118,.124]). The complex mixed-effects linear model on raw RTs resulted in a mean TPR of.856 (95% CI [.853,.859]) and a mean FPR of.092 (95% CI [.090,.095]), while the simple random-effects structure model yielded a mean TPR of.854 (95% CI [.851,.857]) and mean FPR of.092 (95% CI [.089,.094]). See Fig. 3 for details.

**Fig. 4:**
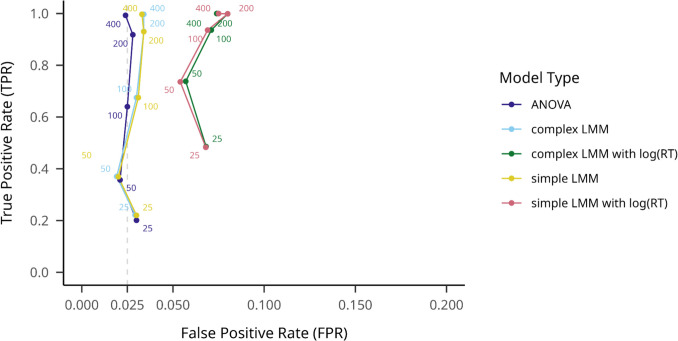
Model TPRs and FPRs on large effect size datasets on different participant numbers, without outlier filtering. Note. True positive rate is indicated on the *y*-axis, while false positive rate is indicated on the *x*-axis. Hypothesis testing models are shown with different colors, and numbers on the plot indicate different sample sizes. An assumed maximum FPR of.025 is indicated with a dashed vertical line

All models yielded considerably lower TPRs and FPRs on non-filtered datasets. On log-transformed RTs, the complex LMM yielded the highest mean TPR of.832 (95% CI [.822,.843]), with an FPR of.070 (95% CI [.063,.077]), while ANOVA resulted in a TPR of.622 (95% CI [.608,.635]) and FPR of.026 (95% CI [.024,.034]). The simple and complex LMMs on raw RTs performed similarly to ANOVA; the complex model yielded TPR of.656 (95% CI [.632,.659]) and FPR of.03 (95% CI [.021,.030]), comparable to the simpler model that yielded a TPR of.839 (95% CI [.625,.652]) and an FPR of.03 (95% CI [.025,.034]). See Fig. 4 for details.

**Fig. 5:**
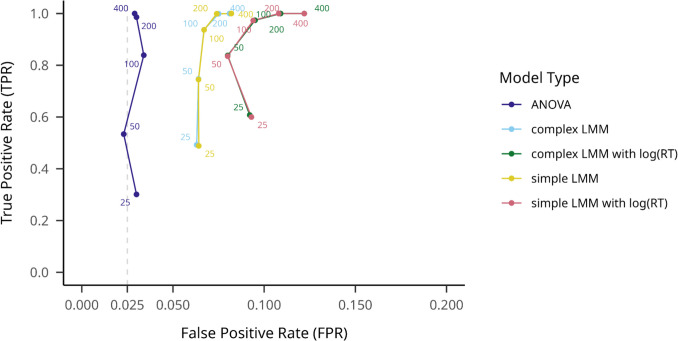
Model TPRs on large effect size datasets on different participant numbers, with the 3SD outlier filtering method. Note. True positive rate is indicated on the *y*-axis, while false positive rate is indicated on the *x*-axis. Hypothesis testing models are shown with different colors, and numbers on the plot indicate different sample sizes. An assumed maximum FPR of.025 is indicated with a dashed vertical line

On the most popular outlier filtering method, the ±3SD distance from the conditional mean method, mean TPRs reflected the aggregated values, with the highest of.884 (95% CI [.875,.893]) for the log(RT) complex LMM, and the lowest of.732 (95% CI [.720,.744]) for the ANOVA. FPRs were slightly inflated compared to the no-filtering condition for the log(RT) complex LMM, at.0998 (95% CI [.091,.108]), and a nominal.029 (95% CI [.025,.034]) for the ANOVA. Details about different sample sizes on different model types are presented in Fig. 5.

#### Comparing outlier filtering methods

**Fig. 6:**
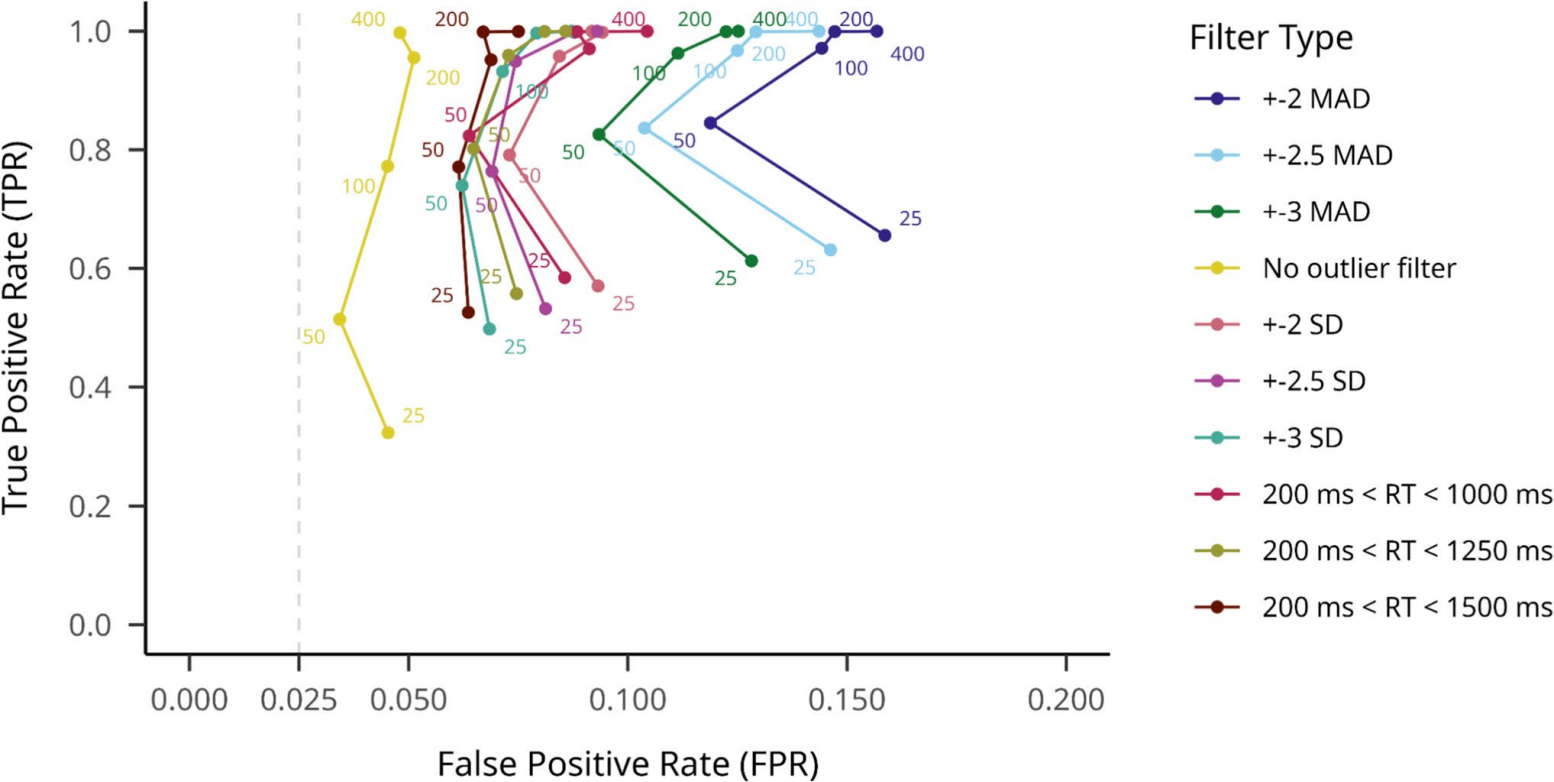
SD-filtering TPRs and FPRs on large effect size datasets across different participant numbers, averaged across hypothesis testing models. Note. True positive rate is indicated on the *y*-axis, while false positive rate is indicated on the *x*-axis. Outlier filtering methods are shown with different colors, and numbers on the plot indicate different sample sizes. An assumed maximum FPR of.025 is indicated with a dashed vertical line

We also compared different outlier filtering methods by aggregating hypothesis testing model results on true effect datasets to calculate TPR, and the results for no effect datasets to calculate FPR (see Fig. 6).

There were substantial differences between the ten filtering methods in overall TPR and FPR means. The no outlier filtering condition resulted in the lowest mean TPR of.714 (95% CI [.709, 719]), but also the lowest mean FPR of.049 (95% CI [.047,.052]). We obtained the highest TPR for the MAD 2.0 method, at.894 (95% CI [.891,.898]); however, this method resulted in the highest FPR of.151 (95% CI [.146,.155]). The 3SD threshold trimming resulted in a mean TPR of.833 (95% CI [.829,.838]) and a mean FPR of.079 (95% CI [.075,.082]).

### Flanker (small-effect) datasets

#### Hypothesis testing models

**Fig. 7:**
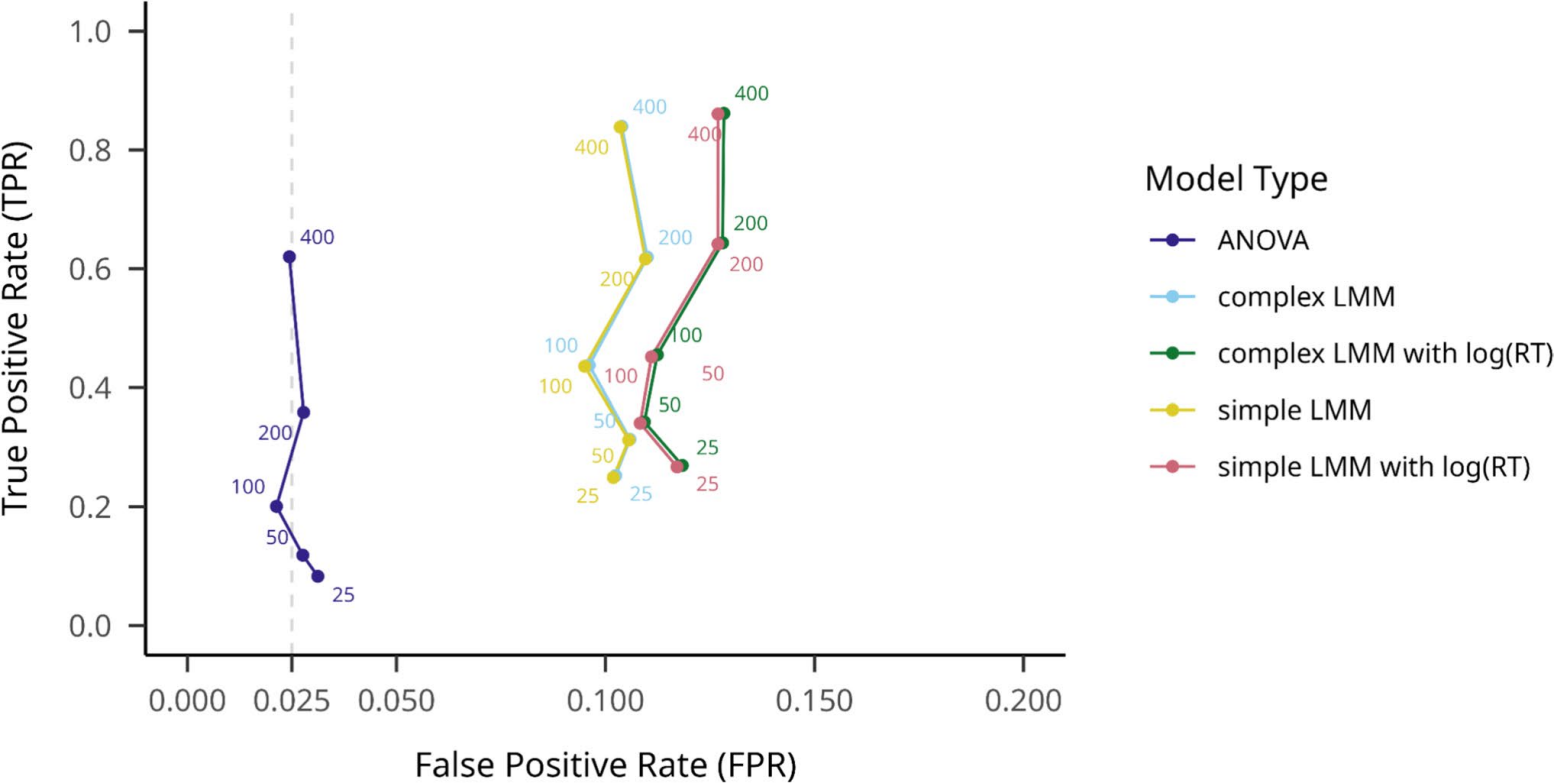
Model TPRs and FPRs on small effect size (flanker) datasets on different participant numbers. Note. True positive rate is indicated on the *y*-axis, while false positive rate is indicated on the *x*-axis. Hypothesis testing models are shown with different colors, and numbers on the plot indicate different sample sizes. An assumed maximum FPR of.025 is indicated with a dashed vertical line

On the small effect size datasets, we first aggregated all outlier filtering methods to average TPRs between the five hypothesis testing models. As shown in Fig. 7, similarly to the large effect datasets, ANOVA performed worst, with a mean TPR of.278 (95% CI [.274,.282]), but with a nominal mean FPR of.026 (95% CI [.025,.028]). The complex LMM on log-transformed RT performed the best, with a mean TPR of.515 (95% CI [.510,.519]), but with a rather high mean FPR of.119 (95% CI [.116,.122]).

**Fig. 8:**
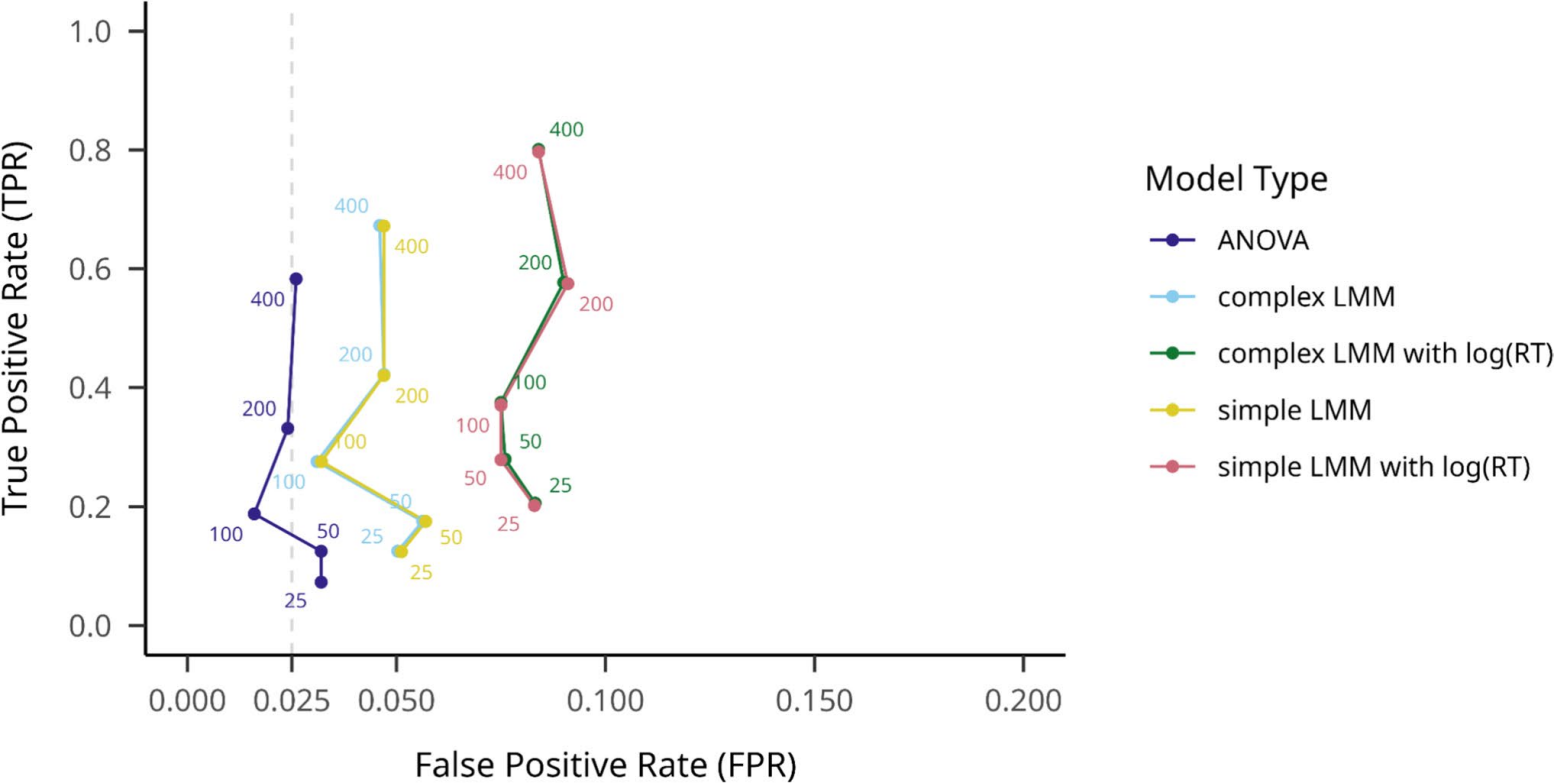
Model TPRs and FPRs on small effect size (flanker) datasets on different participant numbers, with no outlier filtering method used. Note. True positive rate is indicated on the *y*-axis, while false positive rate is indicated on the *x*-axis. Hypothesis testing models are shown with different colors, and numbers on the plot indicate different sample sizes. An assumed maximum FPR of.025 is indicated with a dashed vertical line

When no outlier filtering was applied (see Fig. 8), ANOVA still performed worst on TPR (.261, 95% CI [.249,.273]), but best on FPR (.026, 95% CI [.022,.030]), while the complex log(RT) LMM performed best on TPR (.448, 95% CI [.434,.462]) and worst on FPR (.082 95% CI [.074,.089]).

**Fig. 9:**
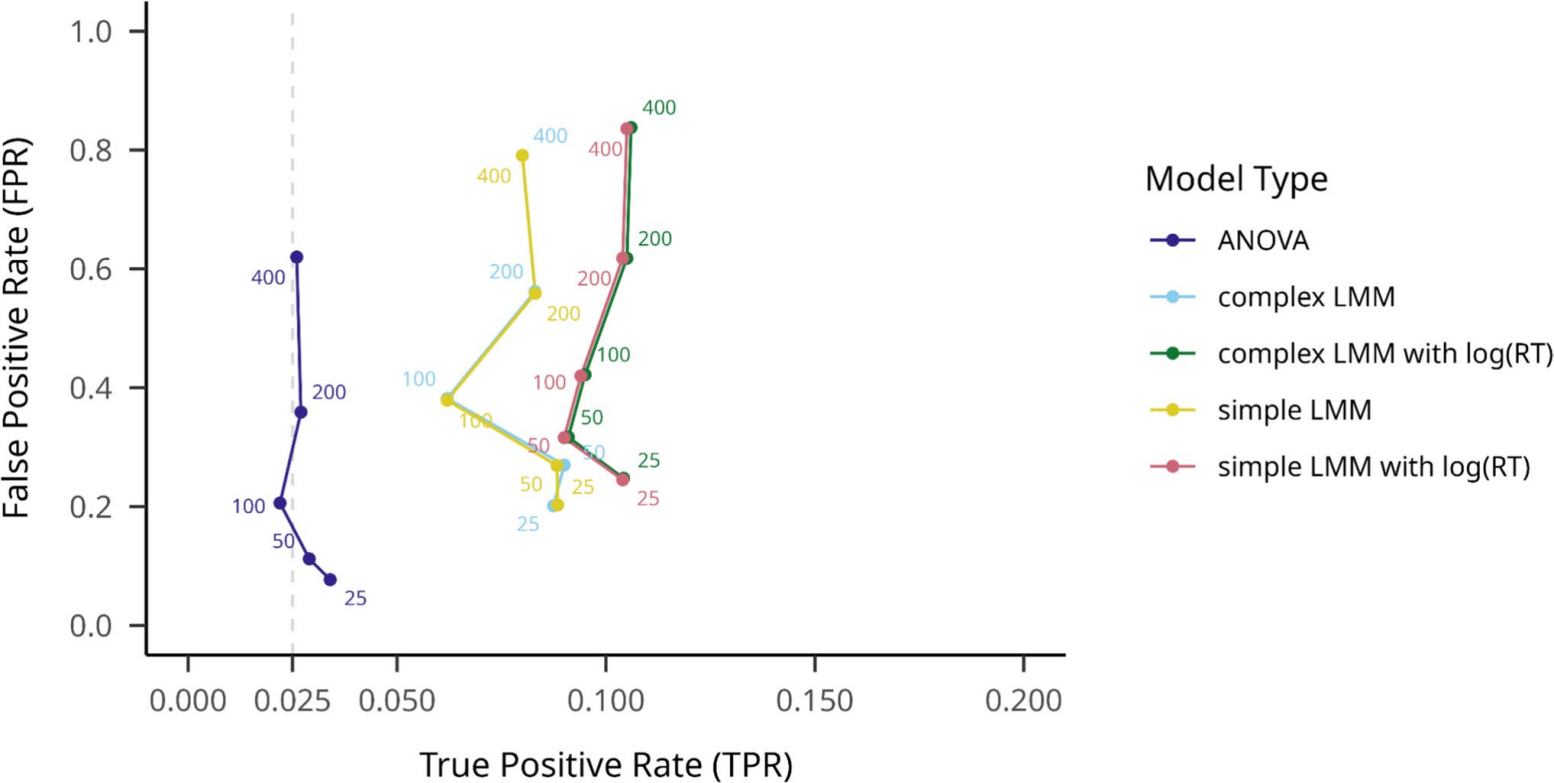
Model TPRs on small *effect size* (flanker) datasets on different participant numbers, with the ±3SD outlier filtering method used. Note. True positive rate is indicated on the *y*-axis, while false positive rate is indicated on the *x*-axis. Hypothesis testing models are shown with different colors, and numbers on the plot indicate different sample sizes. An assumed maximum FPR of.025 is indicated with a dashed vertical line

On the ±3SD outlier filtering method, the results were similar to the averaged results (see Fig. 9): ANOVA, TPR =.275 (95% CI [.262,.287]), FPR =.028 (95% CI [.023,.032]), complex LMM on log-transformed RT, TPR =.489 (95% CI [.475,.503]), FPR =.100 (95% CI [.092,.109]). Overall, all these results are qualitatively similar to those obtained for the large-effect datasets.

#### Comparing outlier filtering methods

**Fig. 10:**
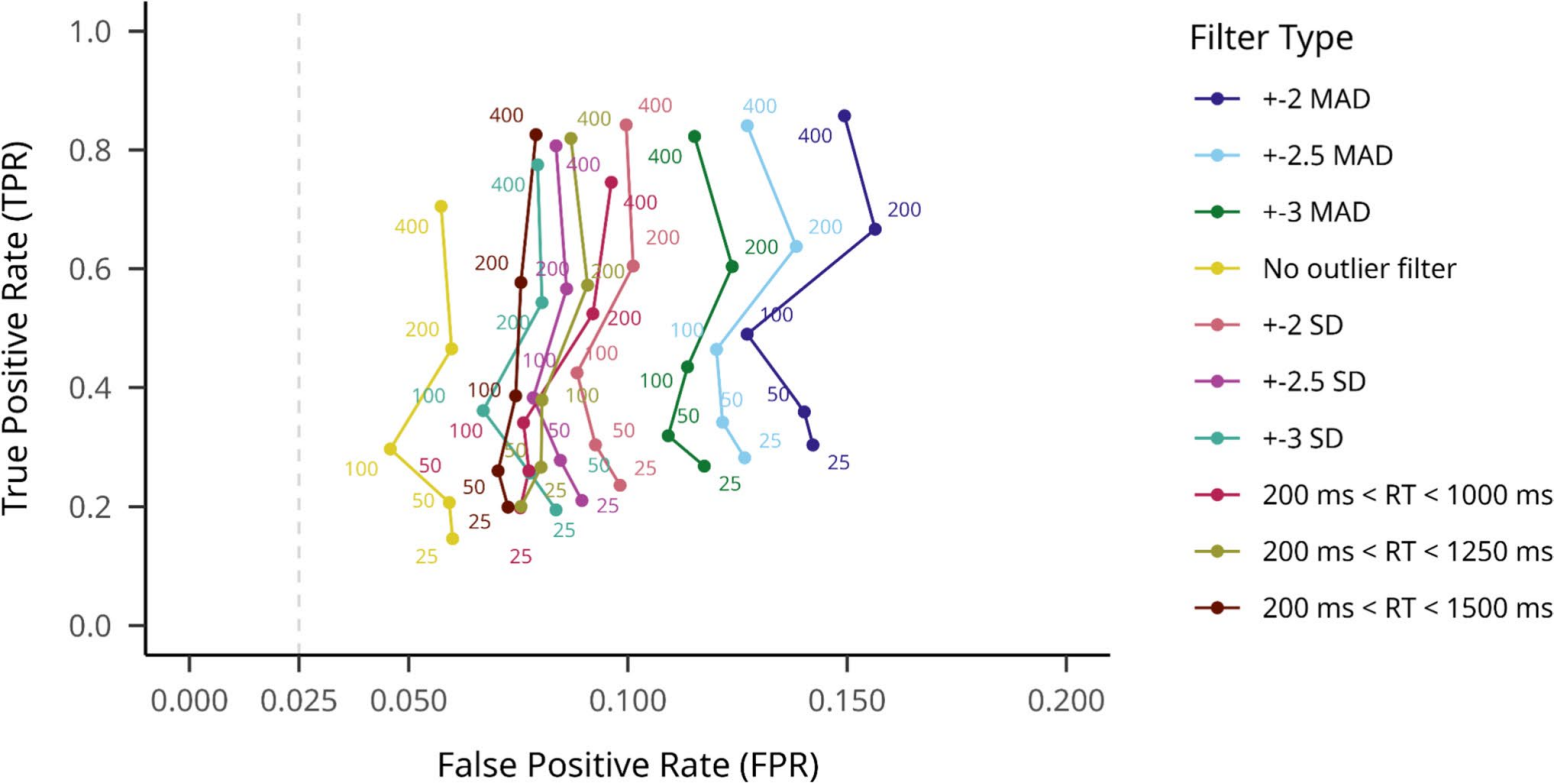
SD-filtering TPRs and FPRs in small effect sizes on different participant numbers averaged across hypothesis testing models. Note. True positive rate is indicated on the *y*-axis, while false positive rate is indicated on the *x*-axis. Hypothesis testing models are shown with different colors, and numbers on the plot indicate different sample sizes. An assumed maximum FPR of.025 is indicated with a dashed vertical line

When we compared outlier filtering methods on small effect size datasets, a similar pattern was visible as in the large effect size analyses (see Fig. 10). The no-outlier-filtering condition resulted in the lowest mean TPR of.365 (95% CI [.359,.371]) and the lowest mean FPR of.062 (95% CI [.059,.065]). We obtained the highest mean TPR for the MAD 2.0 method, at.536 (95% CI [.530,.642]): however, this method resulted in the highest FPR as well:.148 (95% CI [.144,.153]). The 3SD threshold trimming resulted in a mean TPR of.427 (95% CI [.421,.433]) and a mean FPR of.083 (95% CI [.079,.086]).

## Simulation discussion

In this exploratory multiverse simulation, we compared multiple decision pathways in a typical behavioral experimental design to investigate the presence of CSE. To assess the differences in the decision pathways in a scalable manner, we simulated reaction time data based on two tasks, a larger CSE effect size prime-probe task, and a smaller CSE effect size flanker task. Paired with the true effect datasets, we simulated datasets that were equivalent to the above two reaction time data, except without the CSE interaction. We called these prime-probe no-effect, and flanker no-effect datasets. To further increase scalability, sample sizes of 25, 50, 100, 200, and 400 participants were outlined. These two steps resulted in 20 different dataset versions. We generated 1,000 datasets on all dataset versions, resulting in a total of 20,000 individual datasets. We then proceeded to run all chosen outlier exclusion method (10) and hypothesis testing model (five) combinations on all individual datasets, resulting in 1 million data analysis results. To quantify the efficiency of correctly detecting a true effect and incorrectly detecting an effect when it was not present, we measured TPR and FPR on all pathways.

According to the results, the complex LMM fitted to log-transformed RTs, combined with the ±2-MAD outlier filtering yielded the highest TPR; however, this combination also performed the worst on false positives, with a 19.3% FPR (on large effect datasets). Repeated-measures ANOVA with no outlier filtering yielded the lowest TPR, followed by the simple and complex LMMs, on raw RTs. All outlier filtering methods increased the repeated-measures ANOVA’s TPR performance, while FPRs remained around 2.5%. Interestingly, outlier filtering techniques inflated all log-transformed and raw RT models’ FPRs, depending on how strict the given filtering technique was. It is also worth highlighting that log-transformed RT models had FPRs higher than 2.5% (~ 7–8%), even when no outlier filtering method was used, on both effect sizes.

Overall, these simulation multiverse results showed that without outlier filtering methods implemented, linear mixed models did not perform better than repeated-measures ANOVA. Outlier filtering methods could increase statistical power; however, in linear models, these power increases were accompanied by drastic increases in type I errors. Outlier filtering techniques that are based on deviation from the mean or median tend to normalize the otherwise skewed reaction time data, which makes linear models fit better, finding really small mean differences as significant effects. Type I error rates increased with the severity of outlier cutoff points, as the filtering pulled the tails of the distributions closer to the mode of the conditional distribution (see dataset diagnostics section of the supplementary materials). Without any filtering, log transformation of reaction times also normalizes data (as intended), leading to the same overly efficient model fits and inflated type I error rate. The combination of such analytical choices poses the risk of finding CSE in data that does not have the effect. It is worth mentioning that in this simulation we applied rigorous criteria for models to provide evidence for CSE: we did not base conclusions only on *p*-values, but on model fit parameters and estimate directions as well. We accepted only negative estimates as evidence for CSE. The potential allowing of “inverse CSE” would have doubled the amount of false positives provided by linear models. In the most extreme circumstances (very severe outlier filtering combined with log transformation of RTs), non-directional hypothesis testing could have led to nearly 40% false positives. According to the simplest interpretation of CSE, a decrease in congruency effects occurs if the current trial is preceded by an incongruent trial, compared to when it is preceded by a congruent trial (Egner, 2007). In our simulation, the majority of false positive findings were accompanied by substantially smaller estimated raw effect sizes. In our analyses, we did not implement any smallest effect sizes of interest (SESOIs), although defining one would have significantly decreased the number of false positive conclusions. The use of SESOIs, however, is very rare—if not entirely absent—in CSE studies, as theories explaining CSE do not clearly indicate how strong the effect should be, leading to a situation where, in theory, a.5-ms interaction towards the correct direction could mean a significant CSE.

While we cannot recommend one ideal decision pathway based on the above findings, some conclusions can still be made:

- Linear models are only slightly better than repeated-measures ANOVA at finding significant CSE in true effect datasets. This difference is nearly nonexistent when raw unfiltered data is used.
- Outlier filtering methods increase statistical power with the cost of data loss; however, in linear models, type I error rates also increase proportionally to the severity of outlier cutoff points. This inflation of false positive rates poses a significant risk of erroneous findings; therefore, it is not recommended to use linear mixed models along with severe outlier exclusion techniques.
- Type I error rates in repeated-measures ANOVAs were not affected by outlier filtering methods; thus, when severe outlier filtering is justified, repeated-measures ANOVA is a recommended choice for hypothesis testing.
- Outlining the chosen pathway prior to data analysis—for instance, in a preregistration—and staying with it is especially important due to the great variability of possible conclusions different pathways could lead to. Increased family-wise error rate due to multiple testing can get even higher when switching between originally higher FPR pathways.

There are some limitations to the current simulation study. The 50 different analytical pathways investigated in this study are not entirely representative of all the analytical decisions that could be made in a CSE study. There are many other data filtering, data transformation, and hypothesis testing steps that are not covered by this investigation.

Furthermore, the data were generated based on two empirical studies, which were both conducted online, leading to greater than average original noise. First, the specific findings about sample and effect size numbers can only be generalized to these two specific study designs and data structure. Second, these originally noisy datasets were used to fit mixed linear models to calibrate fixed and random effects later used in the data generation, so it is likely that random effects were underestimated in the original models. Third, as the random-effect structure in the generated datasets were defined by the originally fitted models, we could not differentiate between simpler and more complex random-effect structures in the simulation. For both original datasets, the most complex random-effects structure that the linear mixed models could successfully converge on included a random intercept for participants and a random slope for the main effect of congruency. According to the simulations of Barr and colleagues (2013), the maximal level of random-effect structure complexity is recommended to reach maximum power without overly inflating type I error rates. We could not follow this recommendation in our analyses, because the original models could not be fit on the maximal random-effect structure on the obtained prime-probe and flanker datasets. The simulated datasets therefore contained only a simpler random-effect structure consisting of one random slope for congruency and a random intercept for participants. It is worth mentioning, however, that Barr and colleagues (2013) allowed for the progressive simplifying of models in non-convergence scenarios, and performance metrics in these circumstances were also recorded for maximal models. They explained this by the assumption that, when encountering convergence or fit errors, researchers will simplify their model until a point where it converges according to a data-driven approach. We, however, agree more with a theory-driven approach, especially in frequentist statistical frameworks, where fixed and random-effect structures are clearly defined before analyzing or even collecting data. In our simpler simulation, where we assumed a preregistration or registered report environment in which random-effect structures were previously decided, we did not handle convergence and fit errors; instead, we excluded them entirely from the summary analysis. Interestingly, only a few models failed to fit on the simulated datasets.

It is also important to note that the reaction time contaminants we used in this study are likely to be exaggerated and do not necessarily reflect real bad trials. In real life, outliers occur naturally due to factors such as inattentive participants, short disturbances in the experiment, or software or internet issues, probably not in an evenly distributed fashion. The purpose of these additional contaminants was to create excessive noise around the originally clean data generated by the models used for trial simulation. Because of this, the generalizability of the exact sample size and TPR/FPR relationships is limited—given that real datasets might contain much less noise—although differences *between* decision pathway TPRs/FPRs are still comparable.

Another limitation to this study is that the demonstrated simulation is not a perfect representation of the CSE effect. Specifically, to detect underlying effects for the RT distribution, we used the EZ diffusion model, which models data based only on *a*, *v*, and *T*_er_ parameters. Although there are significantly more complex models that could have been used, we decided on this model in order to not overcomplicate the procedure, but still model underlying effects such as the speed–accuracy trade-off. We aimed to generate CSE-related datasets that were as realistic as possible. The goal of this demonstration was to create an accessible simulation, and not to assert general conclusions about the ideal way of analyzing CSE data. The exact results of this simulation are relevant only in situations where the obtained effects used as the basis of the simulation and other distribution and model parameters are similar.

## General discussion

Researchers often face situations where it is not clear how decisions in data analysis should be made in the search for a hypothesized effect. While most data operations are linked to the theory investigated, some operations (such as outlier detection, participant exclusion, or hypothesis testing techniques) are at a researcher’s discretion. The variability in such decisions in published papers assumes a consensus in the research community that all the choices made about these operations are correct and equally viable ways to test a theory. This researcher’s degrees of freedom, however, leads to inconsistency in analytical methods of a scientific field’s literature and even gives space to questionable research practices (e.g., *p*-hacking). If such decisions are indeed equally viable and are associated with equal probabilities of finding the investigated effects, researchers can certainly feel free to make their own choices—provided that those choices are made before first analyzing the data. It is likely, however, that such choices affect the statistical power or type I error of the hypothesis test.

In this study, we demonstrated a multiverse simulation approach that can help to compare different decision pathways used in given fields of research by finding pathways with the highest expected power and lowest expected false positive rates. Specific distributions and effects react differently to data transformations and hypothesis tests; therefore, this approach is also useful for informing a scientific community (e.g., peer reviewers) about the risks of using certain high false positive rate pathways.

Simulation-based power analyses are a common way to estimate the appropriate sample sizes for mixed models (DeBruine & Barr, 2021; Green & MacLeod, 2016), while other sample size and power estimation tools are also widespread for more traditional statistical tests (Faul et al., 2007; Kovacs et al., 2022). These techniques, however, do not consider the different choices researchers make during data analysis. With this exploratory simulation method, the expected power of a hypothesis test could be fine-tuned in order to inform the research community interested in a given effect about best practices in analysis planning. More importantly, an approach like this can highlight risky, low power, or high type I error rate decision pathways, providing information to the scientific community, (e.g., editors or reviewers) when evaluating the analytical choices made in a new study.

General recommendations on reaction time outlier filtering (Berger & Kiefer, 2021), attention checks (Roth & Yakobi, 2024), data transformation (Lo & Andrews, 2015), statistical models (Barr et al., 2013), or other analytical decisions can all be found in the literature, but it is often hard to navigate between a scientific fields’ traditions, and general advice from different fields. The general nature of these recommendations suggests that they should always be right. Still, they may not necessarily apply to a specialized effect, such as the CSE, or other complex interaction effects. Commonly used analytical operations conducted in a specialized field, however, are easily accessible; following them is the most straightforward choice for a researcher. After all, studies are usually reviewed by fellow researchers from a specialized field; thus, following the field’s analytical traditions is the safest bet for every scientist, especially young researchers. Opposing the traditions and deciding along other pieces of advice is often a leap of faith that might also question one’s professional integrity: “*Shall I go with the recommended new method, or stay with the old traditions?”* Or even riskier decisions: *“The old method did not work out. Shall I go with the new method?”* As a compromise between both worlds, an exploratory multiverse simulation such as the present one may help the members of a research field in planning an analytical pipeline with the best sensitivity–specificity ratio, and clear the fog away on the “what-ifs” in psychological data analysis.

## Supplementary Information

Below is the link to the electronic supplementary material.

Supplementary Material 1 (DOCX 86.0 KB)

## Data Availability

All data of the simulation summaries can be found on OSF: https://osf.io/65c4g/files/osfstorage Supplemental materials can be found at: https://osf.io/jcr3t

## Code availability

Code scripts for the simulation can be found on GitHub: https://github.com/mibognar/cse_simulation/tree/hpc

Analysis code can be found at: https://github.com/mibognar/cse_simulation/tree/revision1
