# Supplementary material for: Multiverse simulation to explore the impact of analytical choices on type I and type II errors in a reaction time study
Source: Behav Res Methods. 2025 Sep 18;57(10):291. doi: 10.3758/s13428-025-02807-y (PMC12446153; doi:10.3758/s13428-025-02807-y)
Supplement: Supplementary file 1 — Supplementary Material 1 (DOCX 86.0 KB) [file 13428_2025_2807_MOESM1_ESM.docx]

**Multiverse Simulation to Explore the Impact of Analytical Choices on Type I and Type II errors in a Reaction Time Study - Supplementary materials**

Miklos Bognar^1,2,†,*^*,* Marton A. Varga^2,^*^*^*, Don van Ravenzwaaij^3^, Zoltan Kekecs^2,4^, James A. Grange^5^, Mate Gyurkovics^6^, Balazs Aczel^2^

*^1^Doctoral School of Psychology, ELTE Eötvös Loránd University, ^2^Institute of Psychology, ELTE Eötvös Loránd University, ^3^University of Groningen, Department of Psychology, ^5^School of Psychology, Keele University, ^6^School of Psychology, University of East Anglia*

^†^Correspondence should be addressed to Miklos Bognar; E-mail: [bognar.miklos@ppk.elte.hu](mailto:bognar.miklos@ppk.elte.hu)

^*^Miklos Bognar and Marton Aron Varga are joint first authors

***Literature review***

65 studies were included in the literature review based on PubMed search findings.

**Supplementary Table 1.***Reviewed literature of CSE studies and analytical decisions*

| Authors | Title | outlier detection | hypothesis testing |
| --- | --- | --- | --- |
| Lim, C. E., Cho, Y. S. | Cross-task congruency sequence effect without the contribution of multiple expectancy | RTs<150 ms or SD>2.5 | Repeated measures ANOVA |
| Tang D, Chen X, Li H, Lei Y | Distributional analyses reveal the individual differences in congruency sequence effect | +-3SD | Repeated measures ANOVA and LLM |
| Giacomo Spinelli , Stephen J. Lupker | Conflict-monitoring theory in overtime: Is temporal learning a viable explanation for the congruency  sequence effect? | no outlier filtering mentioned | Anova, GLMM and Bayes factor analyses: random intercepts for participants and items |
| Tomat, M., Wendt, M., Luna-Rodriguez, A., & Jacobsen, T. | Adjustments of selective attention to response conflict - controlling for perceptual conflict, target-distractor identity, and congruency level sequence pertaining to the congruency sequence effect | Rts<200 ms or >2500 ms | Repeated measures ANOVA |
| Daniel R. Westfall, Shih-Chun Kao, Mark R. Scudder, Matthew B. Pontifex, Charles H. Hillman | The association between aerobic fitness and congruency sequence effects in preadolescent children | +-3SD | Repeated measures ANOVA |
| Fröber, K., & Lerche, V. | Performance‑contingent reward increases the use of congruent distracting information | +-3SD | Repeated measures ANOVA |
| Rodriguez-Raecke R, Schrader C, Tacik P, Dressler D, Lanfermann H, Wittfoth M | Conflict adaptation and related neuronal processing in Parkinson’s disease | no outlier filtering mentioned | ANCOVA |
| Gyurkovics, M., Kovacs, M., Jaquiery, M., Palfi, B., Dechterenko, F., & Aczel, B. | Registered Replication Report of Weissman, D. H., Jiang, J., & Egner, T. (2014). Determinants of congruency sequence effects without learning and memory confounds | +-2.5SD | repeated measures ANOVA |
| Lowe MS, Buchwald A | Role of cognitive control in resolving two types of conflict during spoken word production | +-2.5MAD | Linear and logistic mixed effects models: random intercept for participant and item, random slopes for block |
| Pekrul, M., Seer, C., Lange, F., Dressler, D., & Kopp, B. | Flanker Task Performance in Isolated Dystonia (Blepharospasm): A Focus on Sequential Effects | Rts<100ms or > 2000 ms;  +-3SD | Repeated measures mixed ANOVA |
| Ye, W., & Damian, M. F. | Effects of conflict in cognitive control: Evidence from mouse tracking | initiation times> 500ms | Linear mixed effects model: Random intercepts for participants were included. |
| Bognar, M., Szekely, Z., Varga, M. A., Nagy, K., Spinelli, G., Hartanto, A., Majeed, N. M., Chen, N. R. Y., Gyurkovics, M., & Aczel, B. | Cognitive control adjustments are dependent on the level of conflict | +-3SD | Repeated measures ANOVA, mixed effects linear regression: Random intercepts for participants, random slopes for current conflict level |
| Grant LD, Cerpa SR, Weissman DH | Rethinking attentional reset: Task sets determine the boundaries of adaptive control | +-3SD | Repeated measures ANOVA |
| Tomat, M., Wendt, M., & Jacobsen, T. | Attentional adjustment in priming tasks: control strategies depend on context | RTs<200 ms or >2500 ms | Repeated measures ANOVA |
| Schiltenwolf M, Kiesel A, Frings C, Dignath D | Memory for abstract control states does not decay with increasing retrieval delays | +-3SD | Bayesian ANOVA |
| Aschenbrenner, A. J., & Balota, D. A. | Dynamic adjustments of attentional control in healthy aging | +-3SD, RTs < 200 ms | Linear mixed effect models: Random intercepts for participants |
| Bräutigam LC, Leuthold H, Mackenzie IG, Mittelstädt V | Exploring behavioral adjustments of proportion congruency manipulations in an Eriksen flanker task with visual and auditory distractor modalities | +-3SD | ANOVA |
| Duthoo W, Abrahamse EL, Braem S, Boehler CN, Notebaert W | The Congruency Sequence Effect 3.0: A Critical Test of Conflict Adaptation | +-2.5SD | Mixed design ANOVA |
| Schmidt, J. R., & Weissman, D. H. | Congruency sequence effects without feature integration or contingency learning confounds | +-3SD | Repeated measures ANOVA |
| Verstynen T. D. | The organization and dynamics of corticostriatal pathways link the medial orbitofrontal cortex to future behavioral responses | +-3SD | One way repeated measures ANOVA, one sample t test, nested linear regression |
| James R. Schmidt, Daniel H. Weissman | Congruency sequence effects and previous response times: conflict adaptation or temporal learning? | no outlier filtering mentioned | Linear mixed effects: Random intercept for participants |
| Sanga Kim, Yang Seok Cho | Congruency sequence effect without feature integration and  contingency learning | Rts<150 ms or >1250 ms | Repeated measures ANOVA |
| Moritz Schiltenwolf, Andrea Kiesel & David Dignath | No Temporal Decay of Cognitive Control in the Congruency Sequence Effect | +-3SD | Repeated measures ANOVA |
| Christopher D. Erba, Andrew J. Aschenbrenner | Multiple expectancies underlie the congruency sequence effect in confound-minimized tasks | no outlier filtering mentioned | Linear mixed effect models: random intercepts for participants were included |
| Weissman, D. H., Jiang, J., & Egner, T. | Determinants of Congruency Sequence Effects Without  Learning and Memory Confounds | +-2.5SD | Repeated measures ANOVA |
| Qian Yang, Gilles Pourtois | Reduced flexibility of cognitive control: reactive, but not proactive control, underpins the congruency sequence effect | Rts<100 ms;  +-3SD | Generalized linear mixed model: Random intercept for each subject |
| Chae Eun Lim, Yang Seok Cho | Response mode modulates the congruency sequence effect in spatial conflict tasks: evidence from aimed‑movement responses | +-3SD | Repeated measures ANOVA |
| Lauren D. Grant,  Daniel H. Weissman | Turning distractors into targets increases the congruency sequence effect | +-3SD | Repeated measures ANOVA |
| Christopher D. Erb, Stuart Marcovitch | Deconstructing the Gratton effect: Targeting dissociable trial sequence effects in children, pre-adolescents, and adults | no outlier filtering mentioned | Mixed ANOVA |
| Daniel H. Weissman, Zoë W. Hawks, and Tobias Egner | Different Levels of Learning Interact to Shape the  Congruency Sequence Effect | +-3SD | Repeated measures ANOVA |
| Lauren D. Grant, Daniel H. Weissman | The Binary Structure of Event Files Generalizes to Abstract Features: A  Nonhierarchical Explanation of Task Set Boundaries for the Congruency Sequence  Effect | +-3SD | Repeated measures ANOVA |
| Chae Eun Lim, Yang Seok Cho | Determining the scope of control underlying the congruency sequence  effect: roles of stimulus-response mapping and response mode | RTs < 150 ms;  +-2SD | Mixed ANOVA |
| Lim EC, Cho SY | Determining the scope of control underlying the congruency sequence effect: roles of stimulus-response mapping and response mode | RTs < 150 ms;  +-2SD | Three way mixed measures ANOVA |
| Zhao L, Bai Y, Ma J, Wang Y. | Local Control Mechanisms of Implicit and Explicit Conflicts | +-2SD | Repeated measures ANOVA |
| Hartmann ME, Gade M, Steinhauser M. | Neural correlates of adaptive cognitive control in working memory | RTs< 200 ms;  +-2.5SD | Repeated measures ANOVA |
| Suarez, I., De los Reyes Aragón, C., Grandjean, A., Barceló, E., Mebarak, M., Lewis, S., … Casini, L. | Two sides of the same coin: ADHD affects reactive but not proactive inhibition in children | RTs< 150 ms;  +-3SD | Repeated measures ANOVA |
| Weissman HD. | Let your fingers do the walking: Finger force distinguishes competing accounts of the congruency sequence effect | +-3SD | Repeated measures ANOVA |
| Dignath D, Kiesel A. | Further Evidence for the Binding and Retrieval of Control-States From the Flanker Task | +-3SD | Repeated measures ANOVA |
| Kim S, Lee HS, Cho SY. | Control processes through the suppression of the automatic response activation triggered by task-irrelevant information in the Simon-type tasks | Rts< 150 ms and > 1250 ms | Repeated measures ANOVA |
| Jeong JH, Cho SY. | The efects of induced and trait anxiety on the sequential modulation of emotional confict | Rts< 150 ms and > 1250 ms | Repeated measures ANOVA |
| Bisset GP, Grant DL, Weissman HD. | Resisting distraction and response inhibition trigger similar enhancements of future performance | +-3SD | Repeated measures ANOVA |
| Brosowsky PN, Crump JCM. | Memory-guided selective attention: Single experiences with conflict have long-lasting effects on cognitive control | no outlier filtering mentioned | Mixed ANOVA |
| Schuch S, Grange AJ. | Increased cognitive control after task conflict? Investigating the N-3 effect in task switching | +-2.5SD | Repeated measures ANOVA |
| Yang Q, Notebaert W, Pourtois G. | Reappraising cognitive control: normal reactive adjustments following conflict processing are abolished by proactive emotion regulation | +-3SD | Repeated measures ANOVA |
| Schroeder PA, Dignath D, Janczyk M. | Individual Differences in Uncertainty Tolerance Are not Associated With Cognitive Control Functions in the Flanker Task | +-2.5SD | Repeated measures ANOVA |
| Luo C, Proctor RW. | A diffusion model for the congruency sequence effect | +-3SD | Repeated measures ANOVA |
| Weissman DH, Colter K, Drake B, Morgan C. | The congruency sequence effect transfers across different response modes | +-3SD | Repeated measures ANOVA |
| Li N, Wang Y, Jing F, Zha R, Wei Z, Yang LZ, Geng X, Tanaka K, Zhang X. | A role of the lateral prefrontal cortex in the congruency sequence effect revealed by transcranial direct current stimulation | Rts<200 ms | Univariate ANOVA & Paired sample t-test & Independent sample t-test |
| Hartmann EM, Gade M, Steinhauser M. | Adaptive control of working memory | RTs < 200 ms;  +-3SD | Repeated measures ANOVA |
| Koob V, Mackenzie I, Ulrich R, Leuthold H, Janczyk M. | The role of task-relevant and task-irrelevant information in congruency sequence effects: Applying the diffusion model for conflict tasks | Rts<100 ms or Rts> 2500 ms;  +-3SD | Repeated measures ANOVA |
| Koob V, Mackenzie I, Ulrich R, Leuthold H, Janczyk M. | The role of task-relevant and task-irrelevant information in congruency sequence effects: Applying the diffusion model for conflict tasks | Rts<100 ms or Rts> 2500 ms;  +-3SD | Repeated measures ANOVA |
| Li Z, Chen Y, Yin S, Chen A. | Self-referential information optimizes conflict adaptation | +-3SD | Repeated measures ANOVA |
| Duthoo W, Abrahamse EL, Braem S, Notebaert W. | Going, going, gone? Proactive control prevents the congruency sequence effect from rapid decay | RTs > 3000 ms;  +-2SD | Repeated measures ANOVA |
| Larson MJ, Clayson PE, Kirwan CB, Weissman DH. | Event-related potential indices of congruency sequence effects without feature integration or contingency learning confounds | +-3SD | Repeated measures ANOVA |
| Weissman DH, Drake B, Colella K, Samuel D. | Perceptual load is not always a crucial determinant of early versus late selection | +-3SD | Repeated measures ANOVA |
| Scherbaum S, Frisch S, Dshemuchadse M, Rudolf M, Fischer R. | The test of both worlds: identifying feature binding and control processes in congruency sequence tasks by means of action dynamics | +-2.5SD | Repeated measures ANOVA |
| Berger A, Fischer R, Dreisbach G. | It's more than just conflict: The functional role of congruency in the sequential control adaptation | +-3SD | Repeated measures ANOVA |
| Qian Q, Pan J, Song M, Li Y, Yin J, Feng Y, Fu Y, Shinomori K. | Generalization of sequence effects from conflict to cueing tasks | +-2SD | Repeated measures ANOVA |
| Tomat M, Wendt M, Luna-Rodriguez A, Sprengel M, Jacobsen T. | Target-distractor congruency: sequential effects in a temporal flanker task | Rts < 200 ms or Rts > 2500 ms | Repeated measures ANOVA |
| Surrey C, Kretschmer-Trendowicz A, Altgassen M, Fischer R. | Contextual recruitment of cognitive control in preadolescent children and young adults | +-3SD | Repeated measures ANOVA |
| Surrey C, Kretschmer-Trendowicz A, Altgassen M, Fischer R. | Contextual recruitment of cognitive control in preadolescent children and young adults | +-3SD | Repeated measures ANOVA |
| Schuch S, Koch I. | Mood states influence cognitive control: the case of conflict adaptation | +-2SD | Repeated measures ANOVA |
| Schuch S, Pütz S. | Mood state and conflict adaptation: an update and a diffusion model analysis | Rts < 200 ms;  +-4SD | Repeated measures ANOVA |
| Larson MJ, Clayson PE, Keith CM, Hunt IJ, Hedges DW, Nielsen BL, Call VR. | Cognitive control adjustments in healthy older and younger adults: Conflict adaptation, the error-related negativity (ERN), and evidence of generalized decline with age | RT > 1600 ms | Repeated measures ANOVA |
| L. Pires, J. Leitão, C. Guerrini, M. R. Simões | Cognitive control during a spatial Stroop task: Comparing conflict monitoring and prediction of response-outcome theories | RTs < 100 ms;  +-3SD | Repeated measures ANOVA |

***Data simulation and analysis - technical details***

**Supplementary table 2.**

*Original model parameters*

| **Effect Type** | **Parameter** | **Flanker Model – Empirical** | **Flanker Model – Null** | **Prime-Probe Model – Empirical** | **Prime-Probe Model – Null** |
| --- | --- | --- | --- | --- | --- |
| **Fixed Effects** | Intercept | 867.26 | 867.26 | 649.90 | 649.90 |
|  | Congruency | −25.56 | −25.57 | −34.49 | −34.50 |
|  | Previous Congruency | −0.93 | −0.95 | −1.54 | −1.54 |
|  | Congruency × Previous Congruency | −3.65 | −0.01 | −5.21 | 0.01 |
| **Residual Variance** | Residual SD (σ) | 363.01 | 363.04 | 200.98 | 201.05 |
| **Random Effects** | Intercept Variance | 60,778.16 | 60,780.56 | 14,240.63 | 14,237.48 |
|  | Slope Variance (Congruency) | 140.11 | 139.75 | 216.31 | 215.85 |
|  | Intercept × Slope Covariance | −539.05 | −534.47 | 71.98 | 73.32 |

*Effect nullification procedure for null-effect datasets*

The nullification workflow proceeded as follows:

1. Fitted the original (see above) linear mixed effect models predicting reaction time (RT) with trial congruency, trial congruency × previous trial congruency interaction, and participant-level random effects.
2. Stored fitted values, residuals, and variance components from the original models.
3. Interaction nullification
   1. Permuted the interaction terms (*trial congruency × previous trial congruency*) across trials while preserving their marginal distribution
   2. Calculated synthetic RTs (*RT null*) by: (Original fitted values) - (Original interaction effect) + (Null interaction × Original coefficient) + (Original residuals). This removes the systematic influence of the true interaction while preserving other main effects, random effects, and error structure.
4. Re-fit the model using *RT null* to estimate the interaction effect under the null hypothesis, extracting fixed effects, residuals, and variance- covariance components.

*Technical details of the simulation environment*

In our study, we followed a generate-model-analyze cycle. First, we generated data

according to our design and conditions. We saved corresponding identifiers within the generated data to run the matching modeling strategy later. On the generated data, we have fitted models in every condition and saved these model objects as files to analyze them later. To analyze, we've loaded and aggregated relevant parts of the model objects and created a meaningful dataset to make investigation easier. Our computational environment was Atlasz, our university's high performance cluster. The cluster runs on Debian 4.19 x86_64 GNU/Linux. Our R language version was 4.3.2 (2023-10-31). To run R code, we've used the SLURM scheduler (slurm-wlm 18.08.5-2) with the future.batchtools API, enabling us to programatically launch futures (comes from the future R package) as slurm jobs from an R session. The data generation process launched slurm jobs for every condition efficiently, to enable parallelism and faster execution. Similarly, the modeling process loaded the according file and modeled paralelly via slurm jobs. For the analysis, every model was loaded in a separate future, evaluated and filtered, then the relevant results were aggregated in the main R session for a clean dataset.

*Dataframe diagnostics*

In this section we present one dataframe to demonstrate a possible outcome of using different outlier filtering and hypothesis testing techniques.

The ‘small_no_effect_400_0004’ dataset consisted of the reaction time data of 400 simulated participants, each with 400 trials (100 per previous and current trial condition), with a total of 160,000 trials. After excluding error trials generated by the drift diffusion model 147,618 trials remained. As this dataframe was coming from the no-effect simulation pipeline, CSE interaction was nullified in the data and was close to 0 (0.2 ms).

**Supplementary Figure 1.**

*The density of reaction times on the ‘small_no_effect_400_0004’ dataset after error trial exclusions*


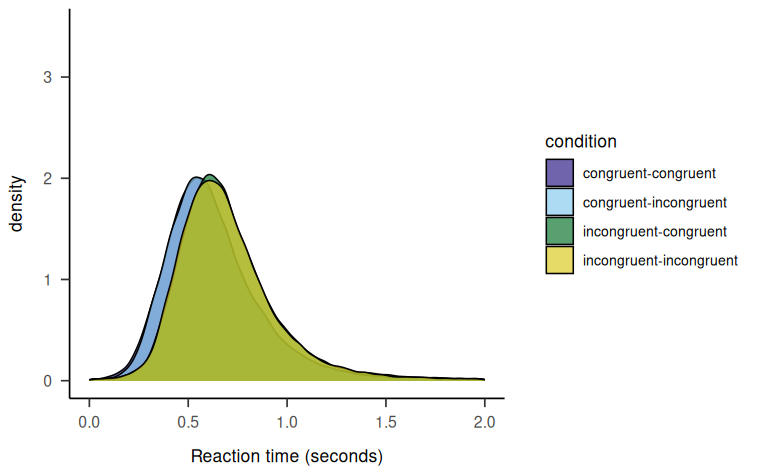


Without further trial exclusions (e.g. outlier filtering) the raw aggregated CSE was -.66ms in the dataset. An ANOVA test of the interaction between previous and current congruency resulted to be nonsignificant, F(1,399) = .003, p = .952. With a mixed-effect linear model where participants were used as random intercept, and current congruency as random slope, the interaction term resulted in a nonsignificant estimate as well, raw estimate = -.192 ms, t(159199) = -.216, p = .829.

When implementing the most severe outlier filtering method in the analytical space investigated in this simulation study, the +-2 MAD method, the aggregated CSE on the filtered dataset was -5.16ms. The ANOVA test of the CSE interaction resulted in a non-significant CSE interaction F(1,399) = 1.9543 p = .164. In contrast, the same linear mixed model described above has found a significant slope for the CSE interaction: raw estimate = -1.32 ms, t(128666) = -3.501, p<.001.

**Supplementary Figure 2.**

*The density of reaction times on the ‘small_no_effect_400_0004’ dataset after error trial and +- 2MAD outlier exclusions*

*
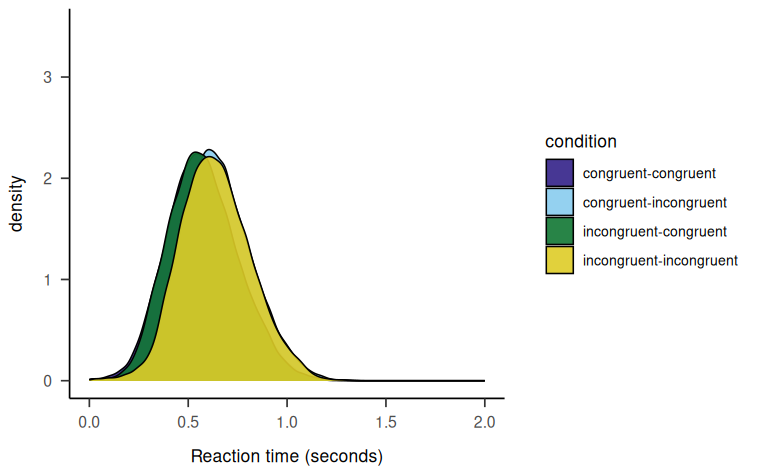
*

In this example a rare, but possible case of false positive outcome is demonstrated. This false positive finding is the result of an accidental inflation of the raw CSE effect in the ‘correct’ direction due to the exclusion of a certain amount of random noise. Additionally to the inflated raw effect, the overall distribution of reaction times were normalized by the trial exclusion procedure making the linear models fit with less error resulting in a significant interaction slope estimate.
